# Supplementary material for: The Blob marine heatwave transforms California kelp forest ecosystems
Source: Commun Biol. 2022 Oct 28;5:1143. doi: 10.1038/s42003-022-04107-z (PMC9614761; doi:10.1038/s42003-022-04107-z)
Supplement: Supplementary file 1 — Supplementary Information [file 42003_2022_4107_MOESM1_ESM.pdf]

The Blob marine heatwave transforms California kelp forest ecosystems

Kristen M. Michaud[1]*, Daniel C. Reed[1], Robert J. Miller[1]

[1]Marine Science Institute, University of California Santa Barbara; Santa Barbara, CA 93117

*Corresponding author. Email: kmmichaud@ucsb.edu

Supplementary Information

Supplementary Figure 1

Supplementary Figure 2

Supplementary Figure 3

Supplementary Figure 4

Supplementary Table 1

Supplementary Table 2

Supplementary Table 3

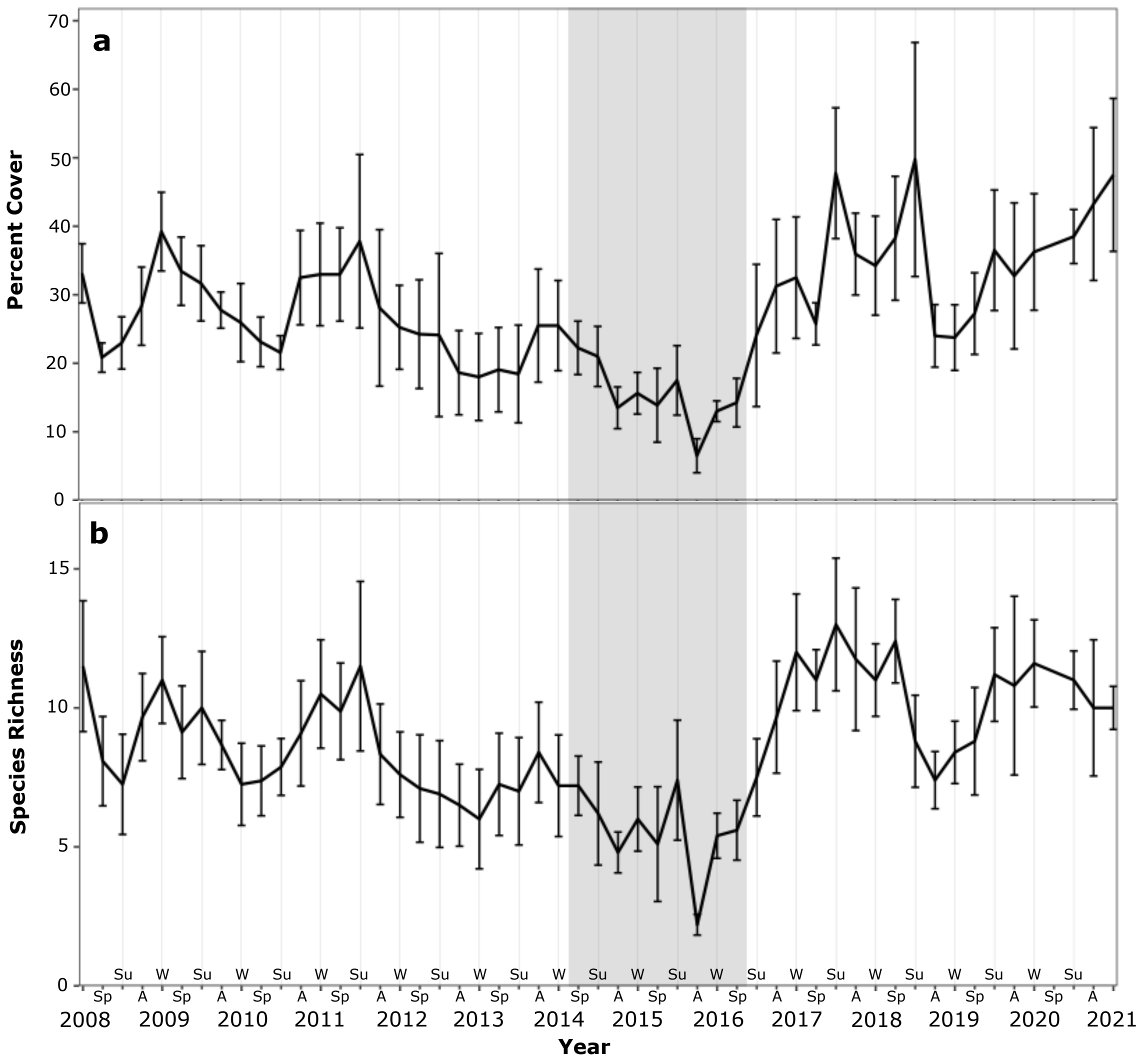

Supplementary Figure 1. Average seasonal a) percent cover and b) species richness of invertebrate phyla across 5 sites. Gray shading indicates anomalous warming period of spring 2014 to the winter of 2016. Missing data during the spring of 2020 corresponds to a data gap due to restrictions on research caused by the COVID-19 pandemic. Bars represent standard error.

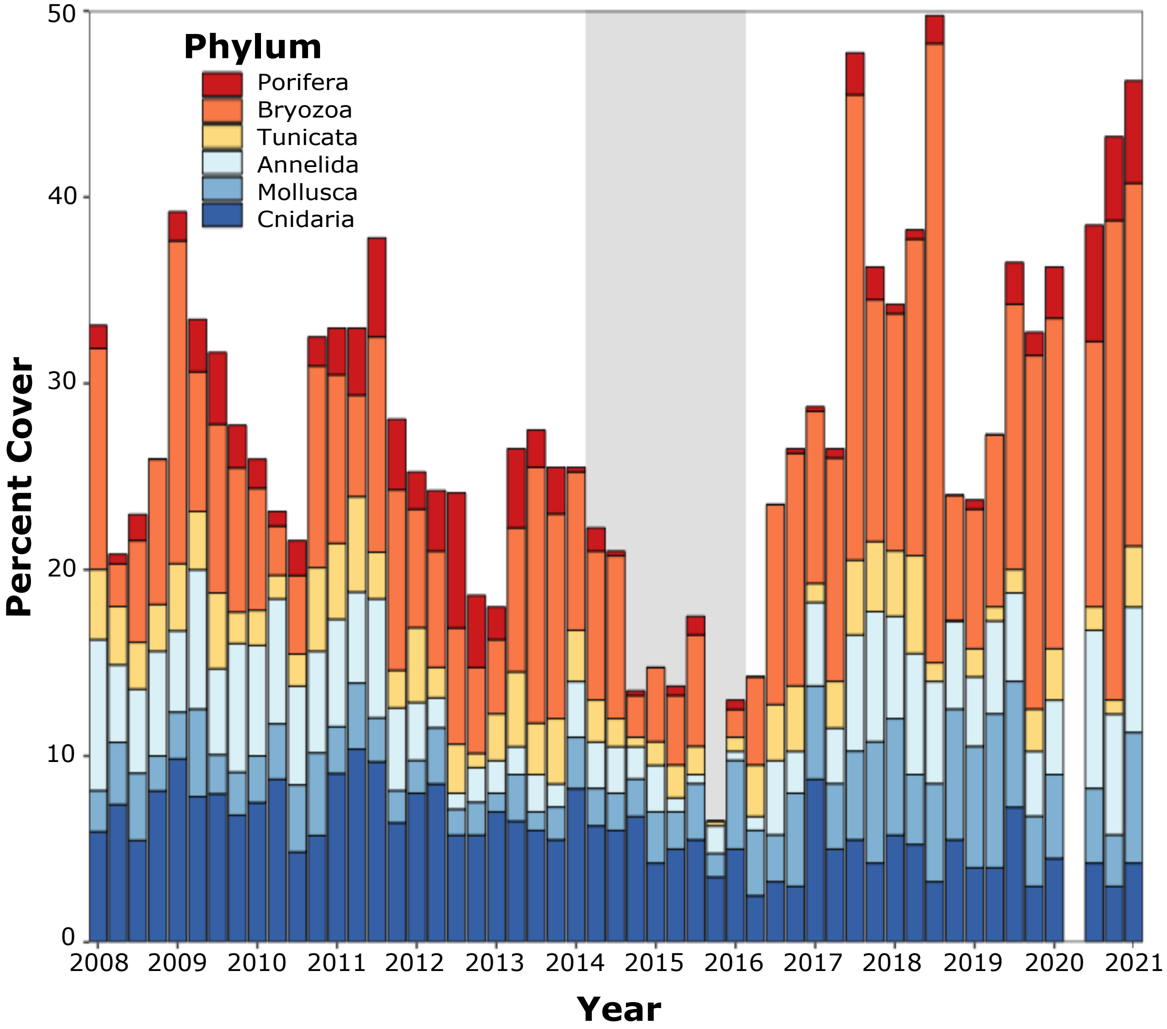

Supplementary Figure 2. Average seasonal percent cover of invertebrate phyla. Gray shading indicates anomalous warming period of spring 2014 to the winter of 2016. Missing data during the spring of 2020 corresponds to a data gap due to restrictions on research caused by the COVID-19 pandemic. Tick marks denote the winter of a respective year.

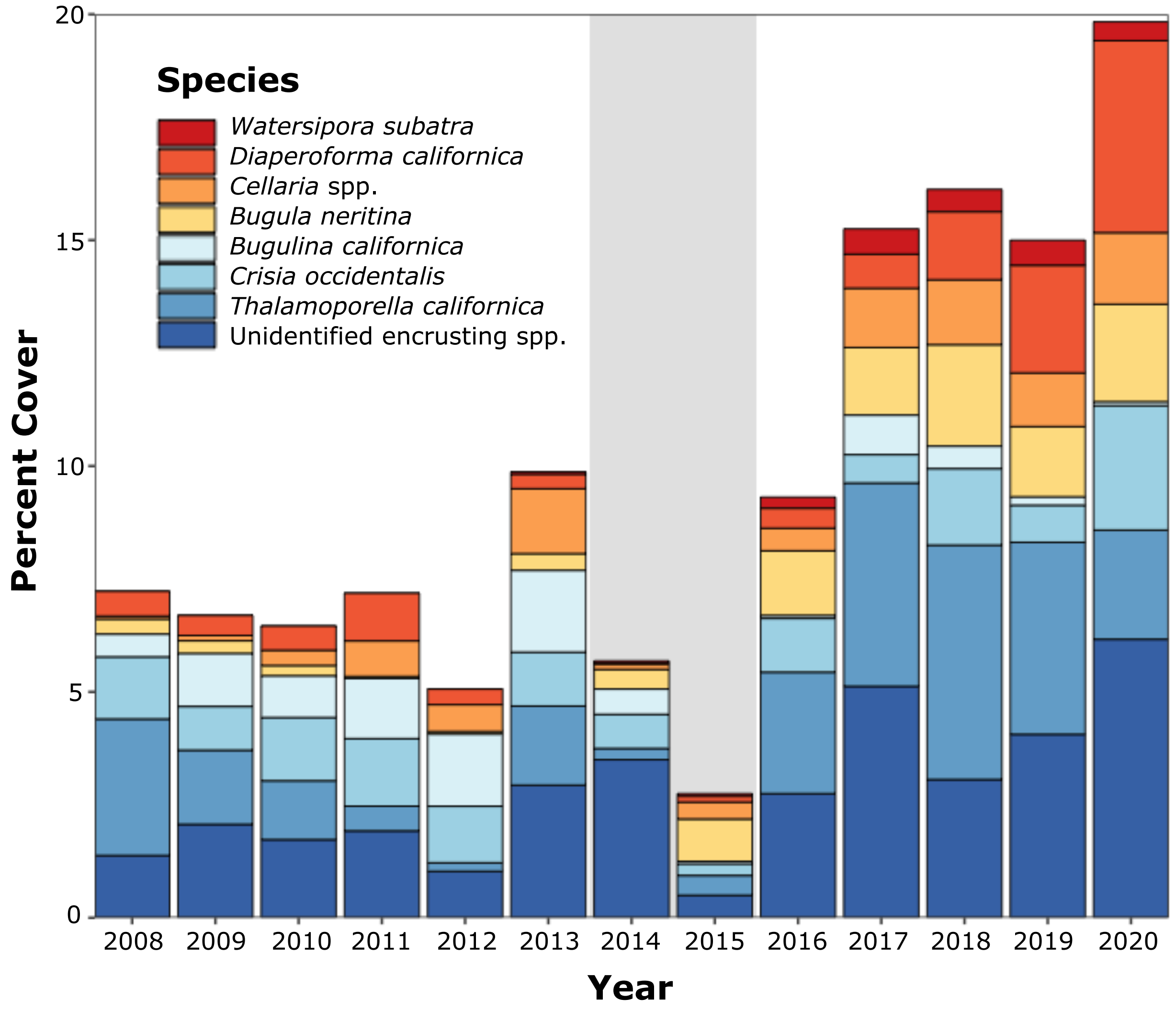

Supplementary Figure 3. **Average annual percent cover of abundant bryozoan species**. The Blob period is highlighted in gray.

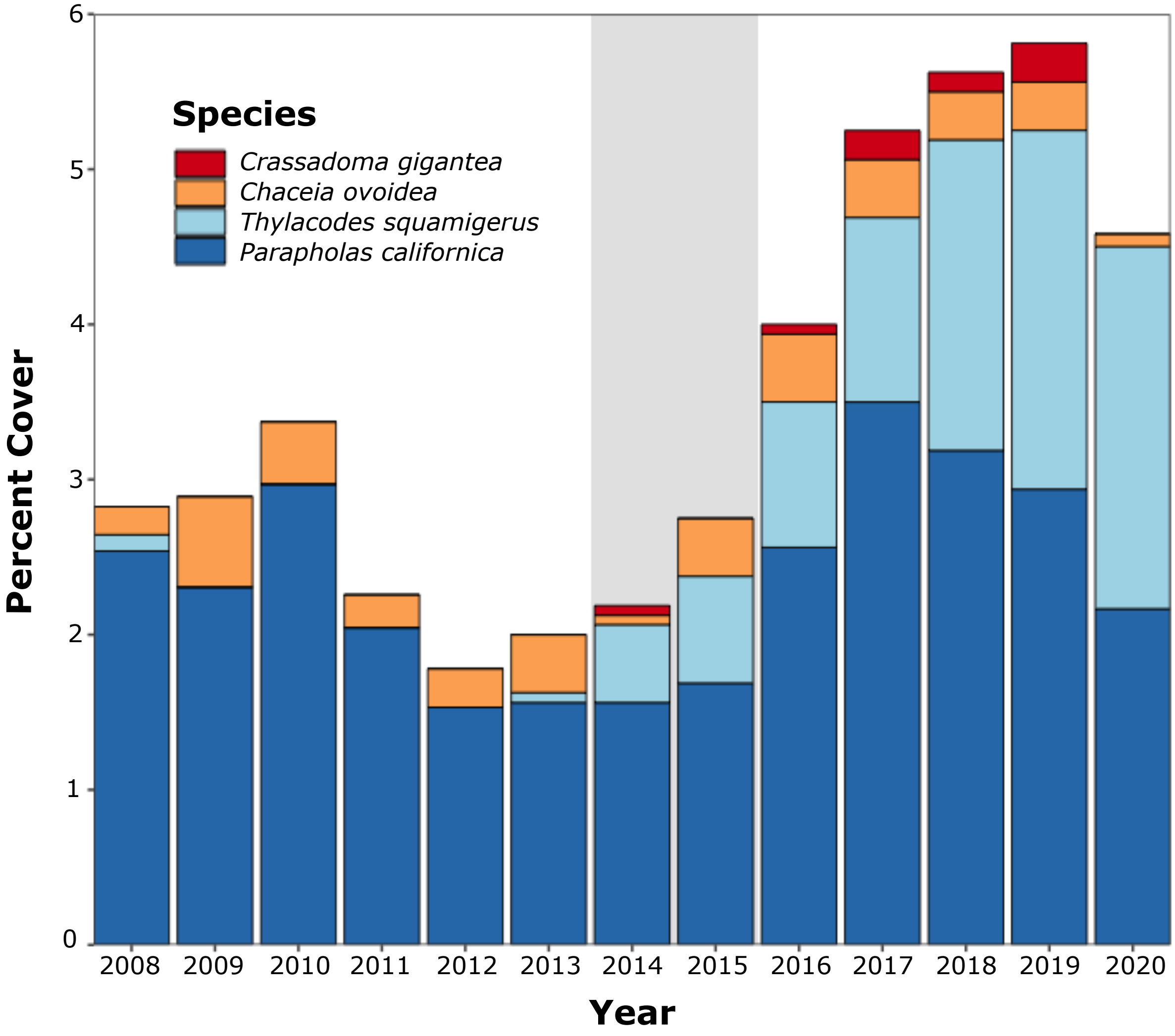

Supplementary Figure 4. Average annual percent cover of abundant mollusk species. The Blob period is highlighted in gray.

Supplementary Table 1.
Sessile invertebrate species that are sampled by the Santa Barbara Coastal Long Term Ecological Research program in point contact surveys.

TAXON	PHYLUM
Diopatra ornata	Annelida
Dodecaceria fewkesi	Annelida
Eudistylia polymorpha	Annelida
Phragmatopoma californica	Annelida
Pista elongata	Annelida
Sabellidae spp.	Annelida
Salmacina tribranchiata	Annelida
Timarete luxuriosa	Annelida
Amathia gracilis	Bryozoa
Bugula neritina	Bryozoa
Bugulina californica	Bryozoa
Cellaria spp.	Bryozoa
Celleporina robertsoniae	Bryozoa
Crisia occidentalis	Bryozoa
Diaperoforma californica	Bryozoa
Heteropora pacifica	Bryozoa
Jellyella tuberculata	Bryozoa
Phidolopora labiata	Bryozoa
Primavelans mexicana	Bryozoa
Thalamoporella californica	Bryozoa
Unidentified Arborescent Bryozoan spp.	Bryozoa
Unidentified Encrusting Bryozoa spp.	Bryozoa
Watersipora subatra	Bryozoa
Chelyosoma productum	Chordata
Clavelina huntsmani	Chordata
Eudistoma psammion	Chordata
Euherdmania claviformis	Chordata
Pycnoclavella stanleyi	Chordata
Styela montereyensis	Chordata
Unidentified Didemnidae spp.	Chordata
Abietinaria spp.	Cnidaria
Actinostella californica	Cnidaria
Agalophenia spp.	Cnidaria
Anthopleura spp.	Cnidaria
Astrangia haimei	Cnidaria
Balanophyllia elegans	Cnidaria
Corynactis californica	Cnidaria
Discophyton rudyi	Cnidaria
Ectopleura spp.	Cnidaria
Halcampa decemtentaculata	Cnidaria
Metridium dianthus	Cnidaria
Muricea californica	Cnidaria
Muricea fruticosa	Cnidaria
Obelia spp.	Cnidaria
Pachycerianthus fimbriatus	Cnidaria
Paracyathus stearnsii	Cnidaria
Plumularia spp.	Cnidaria
Urticina eques	Cnidaria
Chaceia ovoidea	Mollusca
Crassadoma gigantea	Mollusca
Mytilus californianus	Mollusca
Parapholas californica	Mollusca
Thylacodes squamigerus	Mollusca
Acanthancora cyanocrypta	Porifera
Leucilla nuttingi	Porifera
Spheciospongia confoederata	Porifera
Tethya aurantium	Porifera
Unidentified Demospongiae spp.	Porifera

Supplementary Table 2.
Hypothesized paths and directionality of relationships considered in piecewise SEM model (Fig 2). Gray + and – denote possible paths that were not evaluated in the model.

	**Path origin**						
**Path destination**	heatwave days	chl-a	season	kelp	sea urchins	understory macroalgae	invertebrate cover
heatwave days			c [a]				
Chl-a	– [a]		c [a]	–		–	–
season							
kelp	–	–	c [a]		– [a]	–	–
sea urchins	– [a]		c	+		+	+
understory macroalgae	– [a]	–	c [a]	–	–		–
invertebrate cover	– [a]	+ [a]	c [a]	+ [a]	–	– [a]	

[a] Paths that were significant
c Denotes a categorical variable with no directionality

Supplementary Table 3.
Hypothesized paths and directionality of relationships considered in piecewise SEM model (Supplemental Figure 2). Gray + and – denote possible paths that were not evaluated in the model.

	**Path origin**						
**Path destination**	heatwave days	chl-a	season	kelp	sea urchins	understory macroalgae	invertebrate richness
heatwave days			c [a]				
chl-a	– [a]		c [a]	–		–	–
season							
kelp	–	–	c [a]		– [a]	–	–
sea urchins	– [a]		c	+		+	+
understory macroalgae	– [a]	–	c [a]	–	–		–
invertebrate richness	– [a]	+ [a]	c [a]	+ [a]	–	– [a]	

[a] Paths that were significant
c Denotes a categorical variable with no directionality
