# Supplementary material for: The Blob marine heatwave transforms California kelp forest ecosystems
Source: Commun Biol. 2022 Oct 28;5:1143. doi: 10.1038/s42003-022-04107-z (PMC9614761; doi:10.1038/s42003-022-04107-z)
Supplement: Supplementary file 2 — Reporting Summary-New [file 42003_2022_4107_MOESM2_ESM.pdf]

nature portfolio

Corresponding author(s): Kristen Michaud
Last updated by author(s): 09/28/2022

Reporting Summary

Nature Portfolio wishes to improve the reproducibility of the work that we publish. This form provides structure for consistency and transparency in reporting. For further information on Nature Portfolio policies, see our Editorial Policies and the Editorial Policy Checklist.

Statistics

For all statistical analyses, confirm that the following items are present in the figure legend, table legend, main text, or Methods section.

n/a	Confirmed	
☐	☒	The exact sample size (n) for each experimental group/condition, given as a discrete number and unit of measurement
☐	☒	A statement on whether measurements were taken from distinct samples or whether the same sample was measured repeatedly
☐	☒	The statistical test(s) used AND whether they are one- or two-sided<br>*Only common tests should be described solely by name; describe more complex techniques in the Methods section.*
☐	☒	A description of all covariates tested
☐	☒	A description of any assumptions or corrections, such as tests of normality and adjustment for multiple comparisons
☐	☒	A full description of the statistical parameters including central tendency (e.g. means) or other basic estimates (e.g. regression coefficient) AND variation (e.g. standard deviation) or associated estimates of uncertainty (e.g. confidence intervals)
☐	☒	For null hypothesis testing, the test statistic (e.g. F, t, r) with confidence intervals, effect sizes, degrees of freedom and P value noted<br>*Give P values as exact values whenever suitable.*
☒	☐	For Bayesian analysis, information on the choice of priors and Markov chain Monte Carlo settings
☒	☐	For hierarchical and complex designs, identification of the appropriate level for tests and full reporting of outcomes
☒	☐	Estimates of effect sizes (e.g. Cohen's d, Pearson's r), indicating how they were calculated

Our web collection on statistics for biologists contains articles on many of the points above.

Software and code

Policy information about availability of computer code

Data collection	Code to extract chlorophyll-a concentrations at each site is publicly available at:<br>Michaud, K. M. (2022). kristenmichaud/Chl-File-Code-Michaud-et-al: Chl_file_code_Michaud_et_al (Version v1). Zenodo. https://doi.org/10.5281/zenodo.7117750<br>Code for data analysis is publicly available and can be accessed at: Michaud, K. M. (2022). kristenmichaud/Community-Analysis-Michaud-et-al: Community_Analysis (Version v1). Zenodo. https://doi.org/10.5281/zenodo.7117763
Data analysis	Statistical analyses were performed in R statistical software version 4.1.2 using available packages. Piecewise structural equation models were built using the piecewiseSEM package. Indicator species analyses were performed using the indicspecies package. PERMANOVA using distance matrices were analyzed using the vegan package. Community composition was analyzed using PRIMER-e PERMANOVA+ package version 7. All code is published on GitHub and has an associated DOI on Zenodo.

For manuscripts utilizing custom algorithms or software that are central to the research but not yet described in published literature, software must be made available to editors and reviewers. We strongly encourage code deposition in a community repository (e.g. GitHub). See the Nature Portfolio guidelines for submitting code & software for further information.

Data

Policy information about availability of data

All manuscripts must include a data availability statement. This statement should provide the following information, where applicable:

- Accession codes, unique identifiers, or web links for publicly available datasets
- A description of any restrictions on data availability
- For clinical datasets or third party data, please ensure that the statement adheres to our policy

All data used are available publicly from the Santa Barbara Coastal Long Term Ecological Research program and from NASA (AQUA/MODIS Chlorophyll Data):
SBC LTER: Reef: Bottom Temperature: Continuous water temperature, ongoing since 2000 ver 26. Environmental Data Initiative. https://doi.org/10.6073/pasta/22ed009da1cf41cbf76490ab2c0c5949
SBC LTER: Reef: Long-term experiment: Kelp removal: Cover of sessile organisms, Uniform Point Contact ver 30. Environmental Data Initiative. https://doi.org/10.6073/pasta/1151c1dcf5110432b6d35f7dc00bb834
SBC LTER: Reef: Long-term experiment: Kelp removal: Invertebrate and algal density ver 19. Environmental Data Initiative. https://doi.org/10.6073/pasta/decb1dcc7b35d2ef401b2dd7d79ea257
SBC LTER: Reef: Long-term experiment: biomass of kelp forest species, ongoing since 2008 ver 8. Environmental Data Initiative. https://doi.org/10.6073/pasta/e30eb31ce1f346255910fe17092f00b1
Moderate-resolution Imaging Spectroradiometer (MODIS) Aqua Chlorophyll Data. Available at: doi:10.5067/AQUA/MODIS/L3B/CHL/2018

Field-specific reporting

Please select the one below that is the best fit for your research. If you are not sure, read the appropriate sections before making your selection.

☐ Life sciences ☐ Behavioural & social sciences ☒ Ecological, evolutionary & environmental sciences

For a reference copy of the document with all sections, see nature.com/documents/nr-reporting-summary-flat.pdf

Ecological, evolutionary & environmental sciences study design

All studies must disclose on these points even when the disclosure is negative.

Study description	Seasonal sampling of five kelp forests was performed along permanent 40m by 2m transects. Benthic community metrics including percent cover and species identity of invertebrates and algae, size and number of giant kelp plants, and density of macroinvertebrates were collected for each reef.
Research sample	Each sample represents benthic community metrics including invertebrate community composition, invertebrate percent cover, species richness, giant kelp biomass, understory algal percent cover, and urchin density per transect during a given season.
Sampling strategy	This study used existing benthic community data collected by the Santa Barbara Coastal Long Term Ecological Research program. As part of a long term experiment, sampling of a "control" transect took place at each site beginning in 2008 and is ongoing. This study evaluated the benthic community control transects over time.
Data collection	Sessile invertebrate and understory macroalgal communities were sampled twice seasonally from 2008 to 2013 and once seasonally from 2013 to winter of 2021. Uniform point contact surveys were performed by divers along a 40m x 2m permanent transect. Paired points were sampled every 0.5m for a total of 80 points per transect. Sessile invertebrates and algae were identified to species or family level. All species intersecting the point were recorded, such that the total percent cover of all species combined could exceed 100%, while the cover of any individual species could not exceed 100%. The total number of unique species encountered in 80 points per transect was used to measure sessile invertebrate species richness. Giant kelp (Macrocystis pyrifera) was sampled along the same permanent transect and the total number of plants and stipes per plant were recorded and converted to biomass using established allometric relationships. The density of red (Mesocentrotus franciscanus) and purple (Strongylocentrotus purpuratus) urchins was sampled in six permanent 1m2 quadrats per transect, and the average density across quadrats was calculated for each transect. Bottom temperature at each site was recorded using loggers fixed to the bottom (Stowaway Onset tidbits, Onset Computer, Bourne, Massachusetts, USA). Temperature measurements recorded once every 15 minutes were used to calculate the mean temperature for each season from 2003 through winter of 2021. To calculate the seasonal chlorophyll-a concentration at each site, the average monthly chlorophyll-a concentration was extracted from the mapped chlorophyll data from the MODIS (Moderate Resolution Imaging Spectroradiometer) instrument aboard the Aqua satellite. Chlorophyll-a values within 4km-square bins were extracted at each site centered 2 km south (offshore) of the reef's location to prevent bin overlap with land. Seasonal chlorophyll-a values were calculated using the average chlorophyll-a value at each reef over three months.
Timing and spatial scale	Sampling occurred seasonally beginning in 2008. Given logistical restrictions regarding field sampling frequency and intensity, 5 local sites were chosen within 30 km of the University of California Santa Barbara campus. All sampling was from small boats using teams of three to four divers per site per day. Each site had a permanent 40m x 2m transect that was sampled once or twice seasonally during earlier years, and once seasonally after 2013.
Data exclusions	Data points were excluded in the event that one community metric was missing -- for example, a sample was excluded if the biomass of giant kelp was not available for a given site during a sampling event. For community composition analyses, all samples from Isla Vista reef were excluded since sampling did not begin until 2011.

Reproducibility	All kelp forest community, temperature, and chlorophyll-a data are publicly available. All sampling methodology is available in detail in data package descriptions from SBC LTER. All code is publicly available on GitHub with associated DOIs on Zenodo.
Randomization	Randomization was not applicable for this study.
Blinding	Blinding was not applicable for this study.

Did the study involve field work? ☒ Yes ☐ No

Field work, collection and transport

Field conditions	Subtidal sampling was conducted from small boats by SCUBA divers. Water temperatures ranged from 55F to 70F for the sampling period. Sampling occured only when ocean conditions were safe enough for small boating and for diving.
Location	The five subtidal reef sites were located along the mainland of the Santa Barbara Channel within the northern portion of the Southern California Bight: Carpinteria (119.54W, 34.39 N), Mohawk (119.73 W, 34.39 N), Isla Vista (119.86 W, 34.40 N), Naples (119.95 W, 34.42 N), and Arroyo Quemado (120.12 W, 34.47 N). Sites ranged in depth from 5.4 to 7.5m on rocky substrate (shale, sandstone, mudstone).
Access & import/export	All sites were accessed using small boats. Anchors were dropped in sandy patches to avoid disturbing the reef whenever possible.
Disturbance	Divers made efforts to minimize the impact of sampling on the benthic community by not removing any organisms on or near the fixed transect. Permanent bolts were fixed to the bottom to mark the transect every 8 meters.

Reporting for specific materials, systems and methods

We require information from authors about some types of materials, experimental systems and methods used in many studies. Here, indicate whether each material, system or method listed is relevant to your study. If you are not sure if a list item applies to your research, read the appropriate section before selecting a response.

Materials & experimental systems

n/a	Involved in the study	
☒	☐	Antibodies
☒	☐	Eukaryotic cell lines
☒	☐	Palaeontology and archaeology
☒	☐	Animals and other organisms
☒	☐	Human research participants
☒	☐	Clinical data
☒	☐	Dual use research of concern

Methods

n/a	Involved in the study	
☒	☐	ChIP-seq
☒	☐	Flow cytometry
☒	☐	MRI-based neuroimaging
